# Nonintubated minimally invasive chest wall stabilization for multiple rib fractures: a prospective, single-arm study

**DOI:** 10.1186/s13017-020-00335-y

**Published:** 2020-09-23

**Authors:** Weigang Zhao, Yonglin Chen, Weiwei He, Yonghong Zhao, Yi Yang

**Affiliations:** 1 Department of Thoracic Surgery, Shanghai Jiao Tong University Affiliated Sixth People’s Hospital, Shanghai, 200233 China; 2 Department of Anesthesiology, Shanghai Jiao Tong University Affiliated Sixth People’s Hospital, Shanghai, 200233 China

**Keywords:** Nonintubated, Minimally invasive, Chest wall stabilization, Rib fracture

## Abstract

**Background:**

Nonintubated video-assisted thoracoscopic surgery has been widely reported in the past decade, while nonintubated chest wall stabilization has not been reported previously. The aim of this study was to evaluate the safety and feasibility of nonintubated minimally invasive chest wall stabilization in patients with multiple rib fractures.

**Methods:**

We conducted a prospective, single-arm, observational study. In this prospective study, 20 consecutive patients with multiple rib fractures were treated using nonintubated minimally invasive chest wall stabilization.

**Results:**

Minimally invasive chest wall stabilization was mostly performed for lateral rib fractures in this study (*n* = 8). The mean operation time was 92.5 min, and the mean blood loss was 49 ml. No patient required conversion to tracheal intubation. The mean extubation time of the laryngeal mask was 8.9 min; the mean postoperative fasting time was 6.1 h; the mean postoperative hospital stay was 6.2 days; the mean amount of postoperative drainage was 97.5 ml; the mean postoperative pain score was 2.9 points at 6 h, 2.8 points at 12 h, and 3.0 points at 24 h; and the mean postoperative nausea and vomiting score was 1.9 points at 6 h, 1.8 points at 12 h, and 1.7 points at 24 h.

**Conclusions:**

Nonintubated minimally invasive chest wall stabilization is safe and feasible in carefully selected patients. Further studies with a large sample size are warranted.

**Trial registration:**

ChiCTR1900025698. Registered on 5 September 2019.

## Introduction

Chest wall stabilization (CWS) has been widely performed in patients with multiple rib fractures worldwide in the past two decades with satisfactory outcomes [1]. It is generally accepted that patients with three or more acutely displaced rib fractures or flail chest should be considered for CWS [2]. However, there are still many patients with multiple rib fractures who refuse to accept CWS. One major concern for the above situation is that the trauma caused by surgery is too great. Under this circumstance, developing new methods to reduce surgical trauma in CWS is very important.

Double-lumen endotracheal tube and one-lung ventilation has been considered a safe and conventional routine methodology for thoracic surgery. However, adverse events such as sore throat, pain, hoarseness, and respiratory complications are not uncommon after conversional intubation and general anesthesia [3, 4]. Compared with general anesthesia with intubation, nonintubated anesthesia can obviously reduce the adverse events described above, a phenomenon that has aroused great interest of thoracic surgeons in the past decade [5–9]. A previous study confirmed that nonintubated video-assisted thoracoscopic surgery (VATS) is safe and feasible for multiple thoracic conditions, without risk of complications caused by conversional intubation or general anesthesia [10].

To further reduce the surgical trauma associated with CWS, some experienced thoracic surgeons have adopted muscle-sparing incisions in CWS to minimize soft tissue injury. Since the major morbidity of CWS is the size of the incision required to perform an open procedure, some surgeons have tried to perform minimally invasive video-assisted rib plating (VARP) in CWS [11]. Compared with traditional CWS, minimally invasive CWS using the techniques described above can obviously minimize soft tissue injury and reduce the size of incisions.

Based on the results of the studies described above, we believe that nonintubated minimally invasive CWS is esthetically appealing and can obviously reduce surgical trauma. However, no study on nonintubated minimally invasive CWS has been reported until now. Therefore, it remains uncertain whether nonintubated minimally invasive CWS is safe and feasible. In this study, we first report our experience with the use of nonintubated minimally invasive CWS in patients with multiple rib fractures.

## Patients and methods

### Study design and patients

This was a prospective, single-arm, observational study approved by the Ethics Committee of Shanghai Sixth People’s Hospital (approval no: 2019-53). All subjects and/or legal guardians signed informed consent forms in this trial. The study is registered at www.chictr.org.cn (ChiCTR1900025698).

**Fig. 1:**
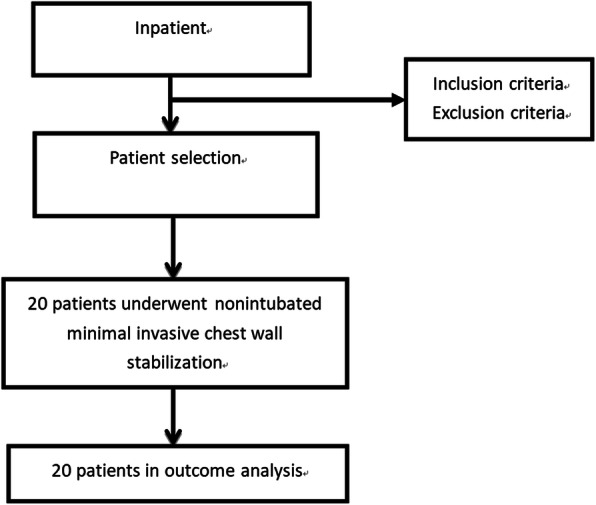
Flow chart of 20 patients screened in this study

Twenty patients who underwent nonintubated CWS for multiple rib fractures in Shanghai Sixth People’s Hospital between June 2019 and September 2019 were included in this study. The inclusion criteria were as follows: (1) American Society of Anesthesiologists (ASA) grade I–II; (2) 18-70 years old; (3) body mass index (BMI) < 28 kg/m^2^; (4) multiple rib fracture patients scheduled for CWS; and (5) preoperative arterial oxygen partial pressure > 60 mmHg and carbon dioxide partial pressure < 50 mmHg. The exclusion criteria were as follows: (1) difficult airway; (2) history of esophageal reflux; (3) myasthenia gravis; (4) coagulation disorders; (5) gastrointestinal ulcers or bleeding; (6) anesthetic drug allergy history; (7) asthma or chronic obstructive pulmonary disease; and (8) pregnant women. The algorithm for patient selection is shown in Fig. 1.

### Anesthetic procedure

**Fig. 2:**
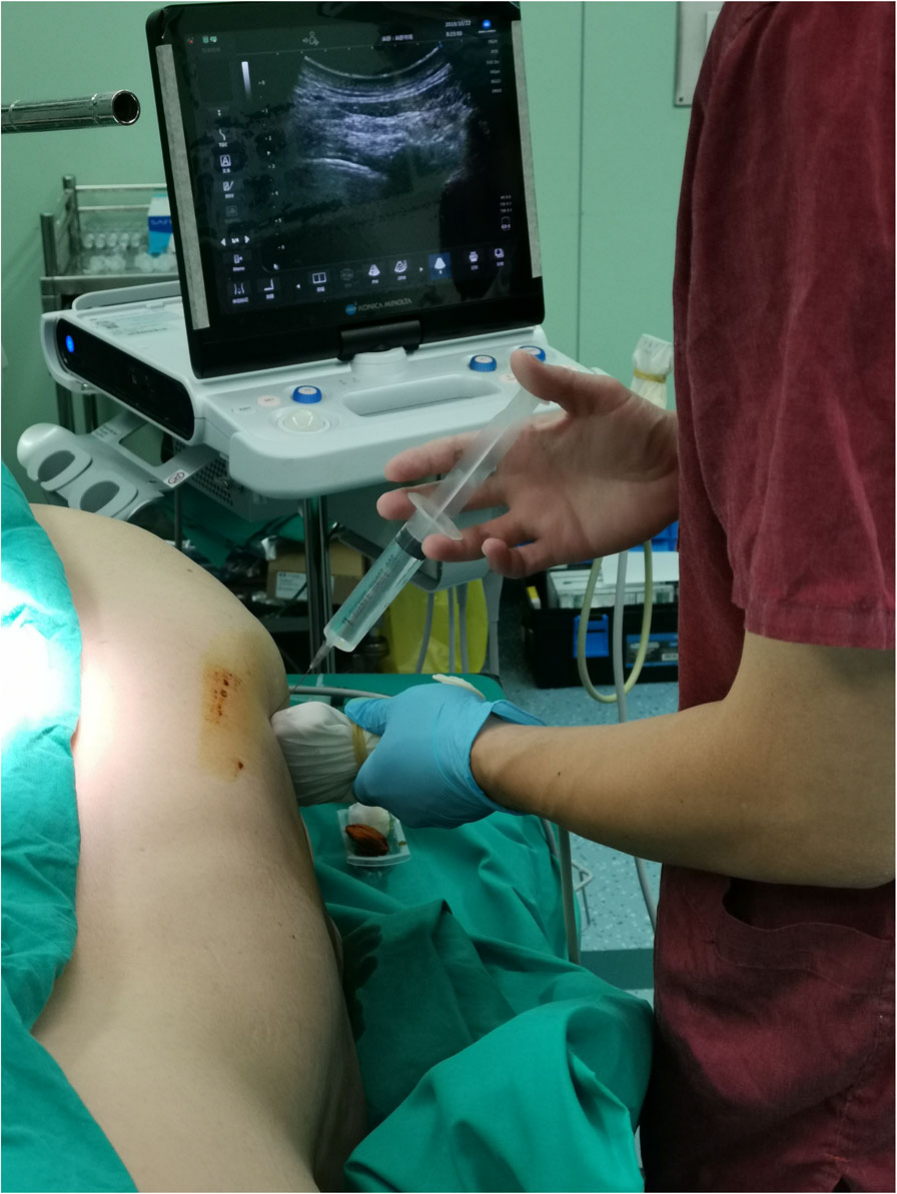
Paravertebral block before anesthesia induction

**Fig. 3:**
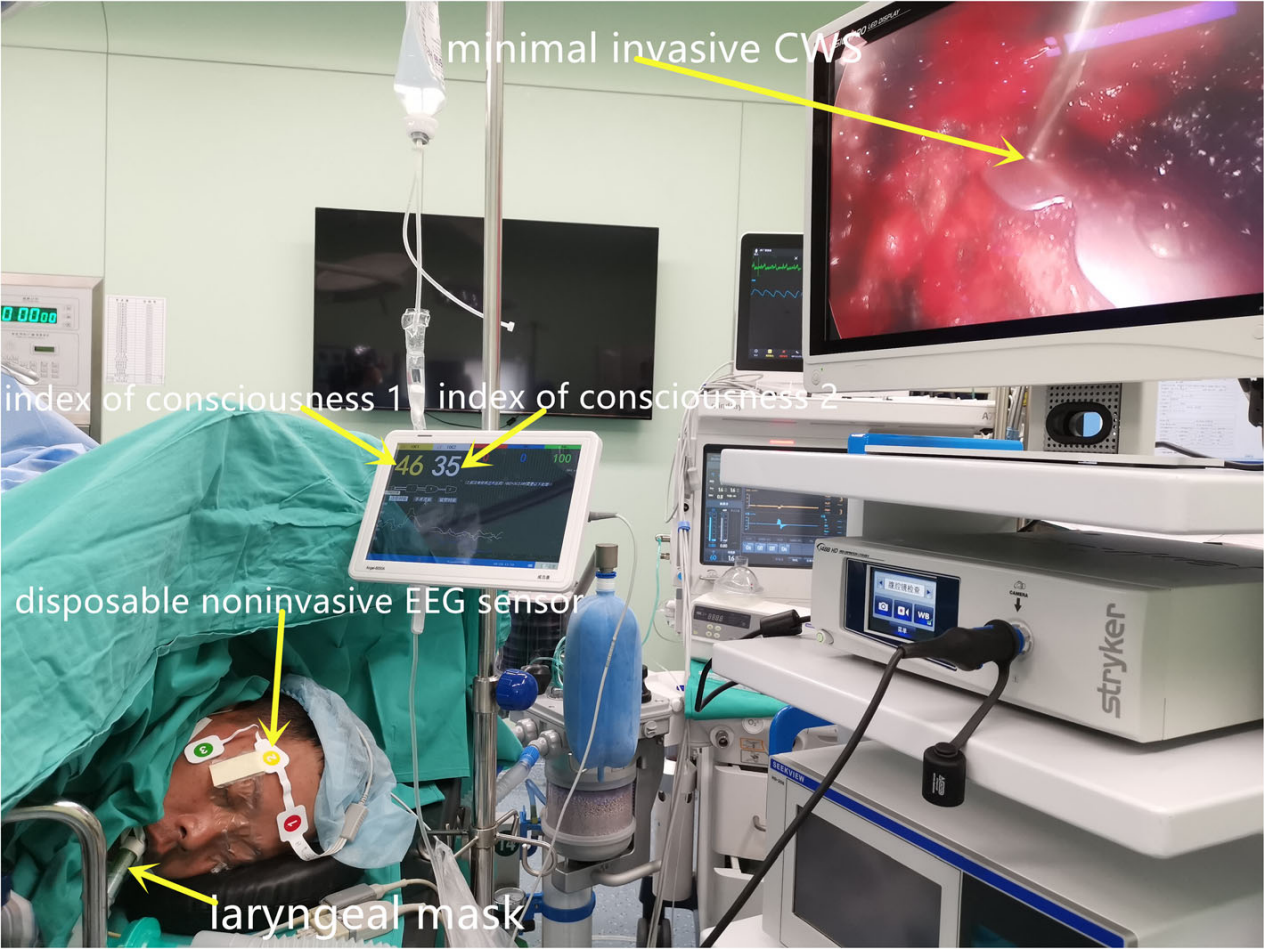
Operative views of a patient undergoing nonintubated minimally invasive chest wall stabilization

Anesthesia was performed by the same team of anesthesiologists. One-shot paravertebral block was performed using 0.4% ropivacaine (3 ml for each segment) before anesthesia induction (Fig. 2). The anesthesia level was ensured to cover the entire operation area. Patients were placed in the lateral position (lateral fractures), supine position (anterior and anterolateral fractures), or posterior decubitus position (posterior fractures). Electrocardiography, noninvasive blood pressure, pulse oximetry, and index of consciousness (IOC) measurements were strictly monitored during the entire process. The IOC is a new anesthesia depth-monitoring indicator. It can be divided into the index of consciousness 1 (IOC1) and the index of consciousness 2 (IOC2). The IOC1 is used to evaluate a patient’s sedation state, while the IOC2 is used to reflect the analgesic depth. Penehyclidine hydrochloride (0.5 mg) and rocuronium (5-10 mg) were given via intravenous injection. Dexmedetomidine hydrochloride (0.3 μg kg^−1^ h^−1^) was given for sedation with a target-controlled infusion pump, and propofol and remifentanil were given using a venous pump. A laryngeal mask airway was used for patients undergoing CWS when an IOC1 value between 40 and 60 was achieved (Fig. 3). Oxygen was delivered by mechanical ventilation (oxygen flow 2 l/min, vital volume 8 ml/kg, breath rate 12 times/min, FiO_2_ = 0.5). Another round of rocuronium (5-10 mg) was given during surgery when necessary. When spontaneous respiration was recovered, mechanical ventilation was stopped, and the oxygen supply was maintained through the anesthesia machine (oxygen flow 2 l/min, FiO_2_ = 0.5).

### Surgical procedure

**Fig. 4:**
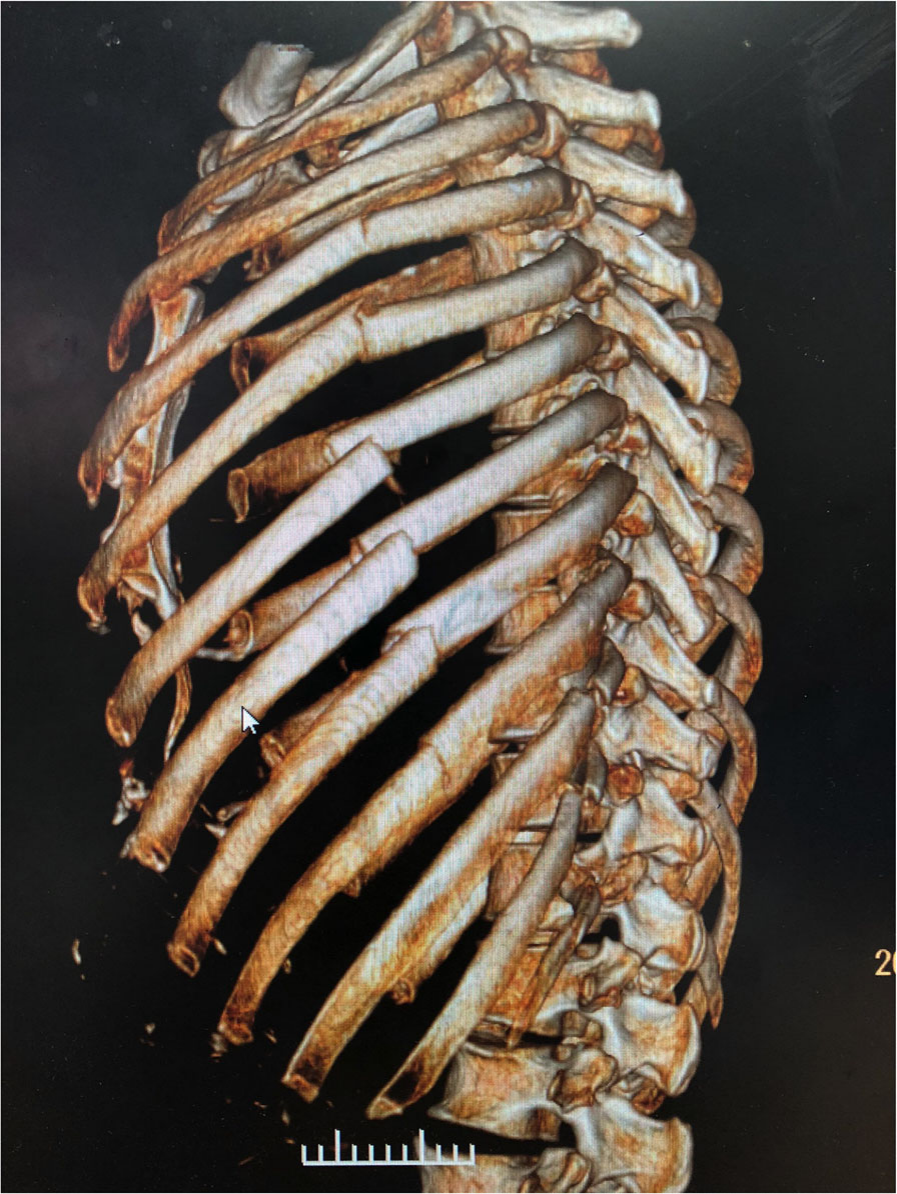
Chest computed tomography and three-dimensional reconstruction of the ribs

**Fig. 5:**
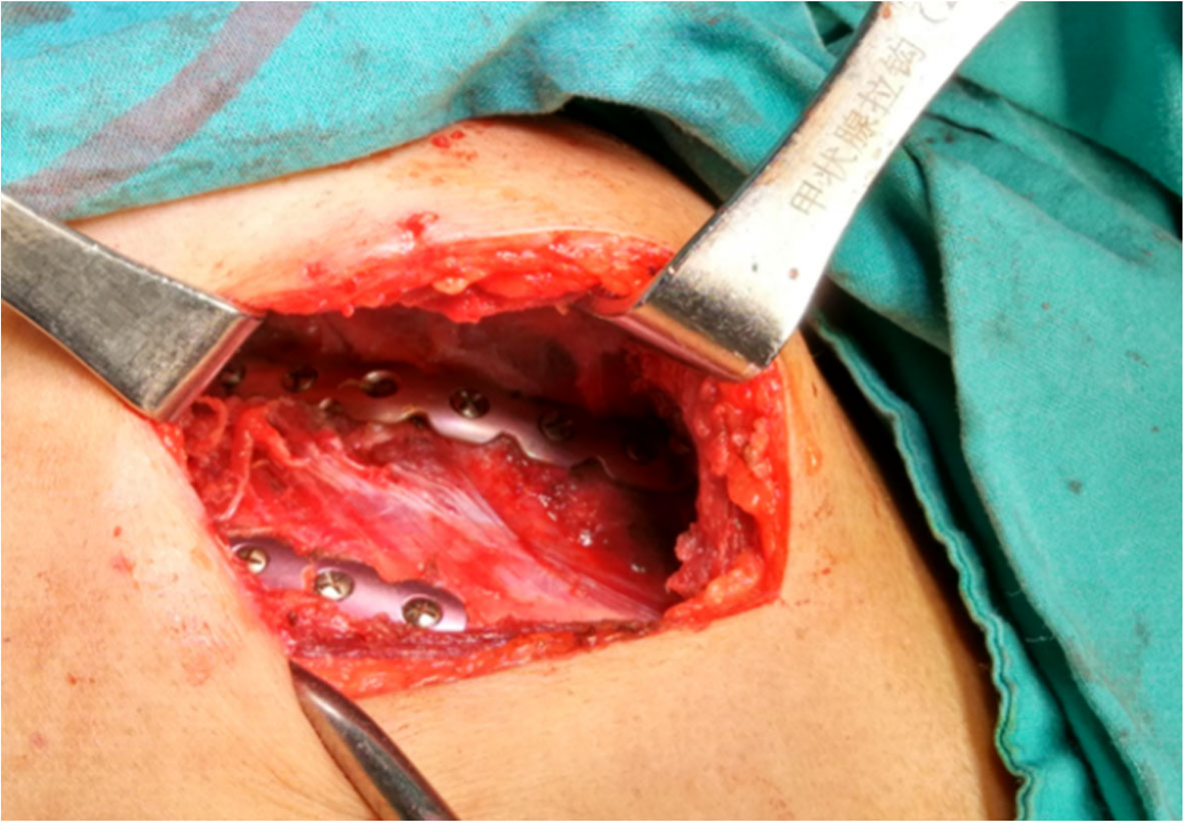
The incision for minimally invasive chest wall stabilization


All operations were conducted by the same team of surgeons. Chest computed tomography and three-dimensional reconstruction of the ribs were performed before surgery to clarify the fracture situation and determine the optimal incision position (Fig. 4). A small incision approximately 5-7 cm in length was made using the muscle-sparing technique (Fig. 5). Anterior rib fractures: an incision was made at the lateral margin of the pectoralis major muscle and the anterior margin of the latissimus dorsi muscle to dissociate the muscles and expose the fracture ends through the muscle gap. Lateral rib fractures: an anterolateral incision was made to dissociate tissue subcutaneously along the anterior iliac muscle fibers to the fracture ends. Posterior rib fractures: an incision was made from the lower edge of the inferior angle of the scapula to the medial edge of the scapula through the auscultation triangle. When dislocated ribs were located in more than one location, more than one incision was made. VARP was performed in some cases with a poor operating field (Additional file 1: Video S1). Postoperative negative pressure drainage was routinely performed to drain the subcutaneous and muscular space fluid in each case, while pleural drainage was performed only when necessary. All displaced fractures of the ribs were fixed.

**Additional file 1: Video S1.** The procedure of video-assisted rib plating.

### Data collection and analyses

We recorded the following baseline characteristics: sex, age, and side, location and number of fractures. SPO2, PaO2, PaCO2, blood pressure (BP), heart rate (HR), vital volume, and breathing rate were recorded at the following time points: preoperatively, intraoperatively, and postoperatively. Operation time, blood loss, and dosage of opioid and vasoactive drugs were recorded during anesthesia. The time of extubation after the operation was also recorded. The visual analog scale (VAS) was used to evaluate pain scores 6 h, 12 h, and 24 h after the operation and postoperative nausea and vomiting (PONV) scores 12 h, 24 h, and 48 h after the operation. All operative and postoperative complications, including anesthesia-related complications, were recorded in this study.

Statistical analyses of the data were performed using SPSS version 23.0 (SPSS Inc., Chicago, IL, USA). Continuous data are described as the means and standard deviations. Categorical data are described as absolute and relative frequencies.

## Results

From June 2019 through September 2019, nonintubated minimally invasive CWS was performed in 20 patients with multiple rib fractures. The characteristics of these patients are reported in Table 1. Among these patients, the mean age was 59.7 years, and 12 patients (60%) were men. CWS was mostly performed for lateral rib fractures in this study (*n* = 8). SpO2, PaCO2, BP, HR, vital volume, and breathing rate were all maintained at normal levels before the operation. The mean pain score was 4.2 points before the operation. The intraoperative results are listed in Table 2. The mean operation time was 92.5 min, and the mean blood loss was 49 ml. No patient required conversion to tracheal intubation. SpO2, PaCO2, BP, HR, vital volume, and breathing rate were also maintained at normal levels during the operation. The postoperative results are listed in Table 3. The mean extubation time of the laryngeal mask was 8.9 min, the mean postoperative fasting time was 6.1 h, and the mean postoperative hospital stay was 6.2 days. Intercostal drainage was needed in only 7 patients, and the mean amount of postoperative drainage was 97.5 ml. Only one patient developed a pulmonary infection after the operation. The mean postoperative pain score was 2.9 points at 6 h, 2.8 points at 12 h, and 3.0 points at 24 h. The mean PONVscore was 1.9 points at 6 h, 1.8 points at 12 h, and 1.7 points at 24 h.

**Table 1:** Clinical characteristics of 20 patients who received nonintubated chest wall stabilization

Characteristic	
Sex	
Male	12 (60%)
Female	8 (40%)
Age	62(27-77) 59.7 ± 12.6
Side	
Right	8 (40%)
Left	12 (60%)
Location	
Anterior^a^	6 (30%)
Lateral^a^	8 (40%)
Posterior^a^	6 (30%)
Number of fractured ribs	5.3 ± 1.1
SPO_2_ (%)	95.9 ± 0.9
PaO_2_ (mmHg)	91.6 ± 10.2
PaCO_2_ (mmHg)	38.1 ± 2.2
Blood pressure (systolic blood press, mmHg)	122.1 ± 9.3
Blood pressure (diastolic blood press, mmHg)	76.3 ± 7.6
Heart rate (times/min)	79.8 ± 8.6
Pain score (VAS)	4.2 ± 0.8
Vital volume (ml)	451.3 ± 25.7
Breathing rate (times/min)	16.8 ± 1.7

^a^If a patient has two or more sites of rib fractures, the site where the fractures are most severe is defined as the fracture site

**Table 2:** Intraoperative results of 20 patients who received nonintubated chest wall stabilization

Variable	
Operation time (min)	92.5 ± 24.7
Blood loss (ml)	49 ± 24.5
Conversion to tracheal intubation	0
Opioid dosage (μg)	12.0 ± 2.5
Dosage of vasoactive drugs	
Norepinephrine (μg)	96.0 ± 30.2
Dosage of muscle relaxants	
Rocuronium (mg)	11.2 ± 4.2
SPO_2_ (%)	97.2 ± 1.2
PaO_2_ (mmHg)	88.6 ± 8.0
PaCO_2_ (mmHg)	38.5 ± 2.8
Blood pressure (systolic blood press, mmHg)	115.2 ± 10.7
Blood pressure (diastolic blood press, mmHg)	71.9 ± 9.6
Heart rate (times/min)	75.4 ± 12.6
Vital volume (ml)	402.5 ± 35.6
Breathing rate (times/min)	13.6 ± 9.0

**Table 3:** Postoperative results of 20 patients who received nonintubated chest wall stabilization

Variable	
Time of extubation after the operation (min)	8.9 ± 2.6
Postoperative fasting time (h)	6.1 ± 1.2
Length of postoperative hospital stay (days)	6.2 ± 1.6
Drainage (ml)	97.5 ± 46.1
Morbidity	
Pulmonary infection	1
SPO_2_ (%)	95.7 ± 0.7
PaO_2_ (mmHg)	93.5 ± 10.5
PaCO_2_ (mmHg)	37.0 ± 2.5
Blood pressure (systolic blood press, mmHg)	121.3 ± 15.2
Blood pressure (diastolic blood press, mmHg)	75.6 ± 9.8
Heart rate (times/min)	76.8 ± 10.9
Vital volume (ml)	453 ± 22.1
Breathing rate (times/min)	17.7 ± 1.6
Pain score (VAS)	
6 h after the operation	2.9 ± 0.7
12 h after the operation	2.8 ± 0.8
24 h after the operation	3.0 ± 0.8
Postoperative nausea and vomiting (VAS)	
12 h after the operation	1.9 ± 0.6
24 h after the operation	1.8 ± 0.5
48 h after the operation	1.7 ± 0.5

*VAS* visual analog scale

## Discussion

Our study is the first to show that nonintubated minimally invasive CWS is safe and feasible in carefully selected patients with multiple rib fractures. Our study showed that the intraoperative and postoperative results were satisfactory. SpO2, PaO2, PaCO2, BP, HR, vital volume, and breathing rate were all maintained at normal levels during and after the operation.

No patient required conversion to tracheal intubation in this study. There are several reasons that may account for the above results. First, a previous study showed that patients with a body mass index of more than 30.0 kg/m^2^ were not suitable for nonintubated anesthesia due to vigorous diaphragmatic and mediastinal movement [5]. Since the body mass index of patients was strictly limited to lower than 28 kg/m^2^ in our study, the risk of conversion to general anesthesia was reduced to the minimum. Second, most procedures involved in CWS did not involve intrathoracic dissection and thus were not easily affected by vigorous diaphragmatic or mediastinal movement.

Only one patient developed a pulmonary infection after surgery. It is generally accepted that patients with multiple rib fractures who are afraid of coughing are likely to develop pulmonary infections. Since postoperative analgesia was satisfactory in all patients, it seems unlikely that patients were afraid to cough because of pain in this study. The cause of the pulmonary infection may be connected with the trauma itself rather than the anesthesia procedure.

Unlike previous studies [5–9], paravertebral block was performed before anesthesia induction in our study. A previous study showed that preemptive pharmacological blockade was effectively used in surgical patients with satisfactory results [12]. It is generally accepted that preemptive analgesia can alleviate pain and stress reactions, maintain hemodynamic stability, reduce intraoperative bleeding, and lower the incidence rates of cardiovascular and cerebrovascular events [13]. In our study, all patients recovered rapidly with satisfactory postoperative analgesia, which supports the further application of preemptive analgesia in CWS.

Compared with video-assisted thoracoscopic surgery, division of thoracic muscles in CWS is more extensive. Although muscle-sparing incisions and minimized approaches can preserve muscles to the maximum extent, dividing muscles is still inevitable in CWS. Mechanical ventilation was necessary in all patients during the first half of the operation since muscle relaxants were used, which made satisfactory oxygenation easy to maintain. The loss of an airway during the procedure in a lateral position is a major complication during the operation. If there is air leakage or changes in the laryngeal mask position in the lateral position, the laryngeal mask can be adjusted in a small range or can be replaced into the larynx; tracheal intubation can also be performed if necessary (no such cases were found in this study). Abdominal distention is a major complication after the operation and is usually caused by air leakage around the laryngeal mask. None of the patients developed abdominal distention in this study, which may be attributed to the appropriate choice of laryngeal mask size and the experience of the anesthesiologist. During the second half of the operation, satisfactory oxygenation was also maintained in all patients. An intact parietal pleura in the majority of cases and the short operation time may have contributed to the above results. VARP is a new technique for CWS that allows for an extrathoracic approach using standard plating assisted by laparoscopy, which likely allows for a faster recovery. A previous study reported their experience with performing the VARP technique and concluded that it was feasible in a cadaver model [11]. Our study is the first to report our experience using the VARP technique in patients with multiple rib fractures with satisfactory results, which supports its application in the general patient population to further define patient indications.

Although no study has been performed before, CWS tends to be safer than video-assisted thoracoscopic surgery. First, CWS usually does not involve intrathoracic procedures, which might avoid triggering coughing in spontaneously breathing patients. Our experience in this study showed that no patient developed cough after nonintubated CWS. Second, hypoxemia and hypercapnia are unlikely to occur in patients without open pneumothorax. Since the parietal pleura was intact in most cases of CWS, it was easy to maintain satisfactory oxygenation during surgery. The satisfactory intraoperative results showed that nonintubated CWS in carefully selected patients was safe and feasible.

Although muscle relaxants were used in all patients, the dosage of muscle relaxants in this study was obviously lower than general anesthesia intubation (10 mg vs. 50 mg). A previous study showed that residual neuromuscular blockade after anesthesia is a cause of increased pulmonary complications such as oxygen desaturation, postoperative pneumonia, airway obstruction, and reintubation [14]. Therefore, it is reasonable to speculate that nonintubated CWS could reduce pulmonary complications even without a control group in this study. Since dividing or dissecting muscles is inevitable in CWS, a low dosage of muscle relaxants may make the whole procedure more difficult. The mean operation time was 87.5 min, which seemed to suggest that the operation procedure did not become difficult under nonintubated anesthesia. Based on the operation, procedure was difficult in patients with posterior rib fractures due to the appearance of muscle tremors during muscle dissection, which suggests that posterior rib fractures might not be the perfect candidates for nonintubated CWS.

The mean extubation time of the laryngeal mask was 8.9 min in this study, which was faster than general anesthesia intubation in our hospital (8.9 min vs. more than 20 min). Several studies have suggested that early extubation can significantly reduce the risk of adverse events such as laryngotracheal injuries, pulmonary hypertensive crises, pulmonary atelectasis, and ventilator-acquired pneumonia [15, 16]. A previous study also showed that early extubation could improve patients’ self-care abilities, accelerate the process of blood circulation in the lungs, and promote more rapid recovery in thoracic surgery [17]. The low incidence of complications after surgery in this study may partly be attributed to the early extubation of the laryngeal mask.

The mean postoperative fasting time was 6.1 h in this study, which was also shorter than general anesthesia intubation in our hospital (6.1 h vs. more than 8 h). It is generally accepted that early postoperative feeding can improve the recovery of gastrointestinal peristalsis and nutritional status and can help patients recover more quickly than traditional postoperative feeding [18]. However, some people worry that early postoperative feeding may result in complications such as pneumonia, aspiration, and mortality. The low incidence of complications after surgery suggested that early postoperative feeding after nonintubated chest wall stabilization was safe and feasible.

There were several inevitable limitations in this study. First, the number of included patients was relatively small. Second, a control group receiving CWS under intubated general anesthesia was lacking in order to compare the differences with nonintubated anesthesia. Considering that this technique had not been performed before, this study had to be designed as a single arm with a small sample size to verify the safety and feasibility of this technique. However, the results of this study support the use of further studies that include a control group with a large sample size.

In our study, all patients showed tolerable postoperative pain and postoperative nausea and vomiting, an early postoperative fasting time, and low morbidity. With the satisfactory results of our study, a prospective study comparing nonintubated anesthesia and general intubated anesthesia in CWS would be helpful to further elucidate the safety and value of nonintubated anesthesia.

## Conclusion

In conclusion, nonintubated CWS is safe and feasible in carefully selected patients. Further studies with a large sample size are warranted to verify the advantages and disadvantages of nonintubated CWS.

## Data Availability

The data that support the findings of this study are available from Shanghai Jiao Tong University Affiliated Sixth People’s Hospital and are available from the corresponding author upon reasonable request.

## Abbreviations

CWS: Chest wall stabilization
VATS: Video-assisted thoracoscopic surgery
VARP: Video-assisted rib plating
ASA: American Society of Anesthesiologists
BMI: Body mass index
PONV: Postoperative nausea and vomiting
VAS: Visual analog scale
IOC: Index of consciousness
MIPO: Minimally invasive plate osteosynthesis
BP: Blood pressure
HR: Heart rate
